# Ten‐year outcomes of whole‐pelvic intensity‐modulated radiation therapy for prostate cancer with regional lymph node metastasis

**DOI:** 10.1002/cam4.5554

**Published:** 2022-12-19

**Authors:** Kiyonao Nakamura, Yoshiki Norihisa, Itaru Ikeda, Haruo Inokuchi, Rihito Aizawa, Toshiyuki Kamoto, Tomomi Kamba, Takahiro Inoue, Toshinari Yamasaki, Shusuke Akamatsu, Takashi Kobayashi, Osamu Ogawa, Takashi Mizowaki

**Affiliations:** ^1^ Department of Radiation Oncology and Image‐Applied Therapy Kyoto University Graduate School of Medicine Kyoto Japan; ^2^ Department of Urology Kyoto University Graduate School of Medicine Kyoto Japan

**Keywords:** lymphatic metastasis, prostate‐specific antigen, prostatic neoplasms, radiotherapy

## Abstract

**Background:**

Management of pelvic node‐positive prostate cancer has been challenging and controversial. We conducted a study to evaluate the outcomes of whole‐pelvic (WP) simultaneous integrated boost (SIB) intensity‐modulated radiation therapy (IMRT) combined with androgen deprivation therapy (ADT).

**Methods:**

A total of 67 consecutive patients with cT1c‐4N1M0 prostate cancer were definitively treated by WP SIB‐IMRT. Neoadjuvant ADT (median: 8.3 months) was administered in all cases. WP SIB‐IMRT was designed to simultaneously deliver 78, 66.3, and 58.5 Gy in 39 fractions to the prostate plus seminal vesicles, metastatic lymph nodes (LNs), and the pelvic LN region, respectively. Adjuvant ADT (median: 24.7 months) was administered in 66 patients.

**Results:**

The median follow‐up period was 81.6 months (range: 30.5–160.7). Biochemical relapse‐free, overall, and prostate cancer‐specific survival rates at 10 years were 59.8%, 79.6%, and 86.3%, respectively. Loco‐regional recurrence was not observed. Being in International Society of Urological Pathology grade group 5 and having a posttreatment detectable nadir prostate‐specific antigen (PSA) level (≥0.010 ng/ml) were significantly associated with worse prostate cancer‐specific survival and progression of castration resistance. The 10‐year cumulative incidence rates of grade 2 and 3 late toxicities were, respectively, 1.5% and 0% for genitourinary, 0% and 1.5% for gastrointestinal events. No grade 4 acute or late toxicities were observed.

**Conclusions:**

WP SIB‐IMRT can be safely administered to patients with pelvic node‐positive prostate cancer. Since grade group 5 and detectable nadir PSA levels are risks for castration resistance, we may need to increase the intensity of treatment for such cases.

## Graphical abstract

Whole pelvic simultaneously integrated boost (WP SIB) IMRT can be delivered safely to patients with pelvic node‐positive prostate cancer. Biochemical relapse‐free, overall, and prostate cancer‐specific survival rates at 10 years were 59.8%, 79.6% and 86.3%, respectively. Being in ISUP grade group (GG) 5 and having a posttreatment detectable nadir prostate specific antigen (PSA) level (≥0.010 ng/ml) were significantly associated with worse prostate cancer‐specific survival and progression of castration resistance.

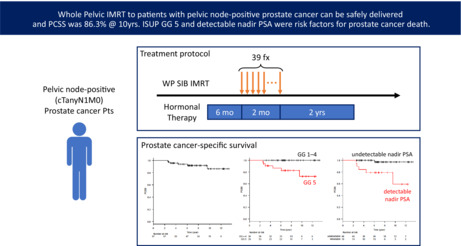

## 1 INTRODUCTION

Among patients diagnosed with prostate cancer, having pelvic node‐positive prostate cancer at the initial diagnosis is relatively rare (approximately 5%–10%).^1^ Patients diagnosed as N1 disease without distant metastases (N1M0) are estimated to be even rarer. Therefore, there have been a limited number of prospective trials regarding N1M0 prostate cancer, and its management has been challenging and controversial. The National Comprehensive Cancer Network (NCCN) guideline for prostate cancer recommends androgen deprivation therapy (ADT) with or without radiation therapy, or radical prostatectomy.^2^

The positive impact of local treatment on survival outcomes in patients with N1M0 disease who were treated with long‐term ADT has recently been shown in a systematic review and a population‐based study.^3^, ^4^ In addition, it was suggested that survival prognoses of N1M0 disease were most favorable among stage IV diseases (cT4N0M0, TxN1M0, and TxNxM1).^5^

It has been shown that a local dose escalation to the prostate significantly improves biochemical and local control rates in external‐beam radiation therapy (EBRT) for localized and locally advanced prostate cancer.^6^ Unfortunately, conventional radiation techniques cannot be used for dose escalation to the prostate when combined with whole‐pelvic (WP) radiation therapy due to the dose limitations of the rectum and bladder. On the other hand, intensity‐modulated radiation therapy (IMRT) allows high‐dose irradiation to the prostate concurrently with pelvic lymph node (LN) irradiation by using the simultaneous integrated boost (SIB) technique.^7^, ^8^

To date, WP IMRT has been applied mainly to high‐risk, localized, and locally advanced cases. There are only two published report regarding WP IMRT for N1M0 prostate cancer.^9^, ^10^ However, in one report, more than half (18/31) of the cases were pN+ patients treated with radical prostatectomy, and in another report more than half of the patients were treated with conventional radiotherapy (RT), not IMRT. There has been no report on clinical outcomes of cN1M0 prostate cancer uniformly treated with high‐dose WP IMRT. Here, we report 10‐year outcomes for 67 patients with cN1M0 prostate cancer treated with high‐dose WP SIB‐IMRT combined with long‐term ADT.

## 2 MATERIALS AND METHODS

Data from 67 consecutive patients with cT1c‐T4N1M0 prostate cancer who were treated with WP SIB‐IMRT were analyzed. All patients provided written informed consent. Data accumulation, pertaining to toxicities and clinical outcomes, was conducted using a follow‐up datasheet at every visit. This study was approved by the local ethics committee (approval number: R1048).

### 2.1 Patients

In our institution, the combination of hormonal therapy and WP SIB‐IMRT, as described below, is applied to patients with N1M0 disease. Between March 2006 and October 2017, 67 patients with cT1c‐4N1M0 prostate cancer, based on the classification of the Union for International Cancer Control (2010; 7th edition), were definitively treated by WP SIB‐IMRT. Pelvic LN metastases were diagnosed based on the presentation of a suspicious LN on computed tomography (CT) and/or magnetic resonance imaging (MRI), which was associated with subsequent shrinkage in size after neoadjuvant ADT (NA‐ADT) on follow‐up imaging. Assuming that the patients in this cohort were clinically N0, the median rate of positive LNs based on the Briganti nomogram was 89% (interquartile range 58.5–100).^11^ M stage was evaluated by CT and bone scan.

The median age was 67.0 years old (range: 51–79), and the initial prostate‐specific antigen (PSA) level was 30.2 ng/ml (4.22–901). Based on the International Society of Urological Pathology Grading system, 4, 4, 25, and 34 patients were classified as grade group (GG) 2, 3, 4, and 5, respectively. GG 2 and 3 are equivalent to Gleason score of 3 + 4 and 4 + 3, respectively, and GG 4 and 5 are equivalent to Gleason score sum of 8 and 9 or higher, respectively.

The patients' characteristics are summarized in Table 1.

**TABLE 1:** Patient characteristics

Number of patients	67
Age (years)	
Median	67
Range	51–79
T stage	
≤T2	13
T3a	17
T3b	21
T4	16
Pretreatment PSA (ng/ml)	
Median	30.2
Range	4.22–901
Grade group	
2	4
3	4
4	25
5	34

Abbreviation: PSA, prostate‐specific antigen.

### 2.2 WP intensity‐modulated radiation therapy

Whole‐pelvic SIB‐IMRT was applied using the dynamic multileaf collimator technique and regular linear accelerators. Simulation procedures were performed as previously reported.^12^ A brief overview of the procedure is as follows. Patients were immobilized in the prone position with a thermoplastic shell, a vacuum pillow, and leg support. Patients were instructed to void their bladder and rectum approximately 1–1.5 h before the CT simulation, according to their individual urinary conditions. CT images of a 2.5‐ or 5‐mm slice thickness were obtained from the lower abdomen to upper thigh.

Radiotherapy treatment planning was performed using the Eclipse treatment planning software (Varian Medical Systems). Target delineations and dose prescriptions for the clinical target volume (CTV) and planning target volume (PTV) for the prostate plus seminal vesicles (CTV_PSV_, PTV_PSV_) were performed in the same manner as the treatment planning for local high‐dose IMRT for patients with locally‐advanced prostate cancer. Therefore, 78 Gy in 39 fractions were prescribed for the PTV_PSV_. The CTV for the elective pelvic LN area (CTV_ELE_) was defined by expanding the iliac arteries and veins by 7 mm. However, skeletal muscles, bones, and intestines were excluded from the CTV_ELE_ if they were included in the expanded volume. The obturator LN areas were included in the CTV_ELE_, but presacral LN areas were not. The PTV_ELE_ was defined by adding a 5 mm margin to the CTV_ELE_. The PTVs for metastatic LNs (PTV_LN_) were defined by expanding the swollen LN size at initial diagnosis by 5‐mm. In the WP SIB‐IMRT plans, 78, 66.3, and 58.5 Gy were delivered simultaneously in 39 fractions as average dose to the PTV_PSV_, PTV_LN_, and PTV_ELE_, respectively. Intestinal doses were limited as follows: the volume of the small intestine receiving ≥60 Gy should be ≤0.5 cc, and the volume of the large intestine receiving ≥65 Gy should be ≤0.5 cc. Doses to the target volumes close to the organs at risk were sacrificed in order to protect the intestinal tract.

### 2.3 Androgen deprivation therapy

Neoadjuvant‐ADT consisted of a 6‐month combined androgen blockade (CAB), which consisted of lutenizing hormone‐releasing hormone (LH‐RH) agonist (goserelin acetate or leuprorelin acetate) plus anti‐androgen (flutamide or bicalutamide); this was planned prior to IMRT in our EBRT protocol for localized prostate cancer. However, there were variations in the duration of NA‐ADT, because a number of patients in whom ADT had commenced several months earlier were referred from outside our institution. In addition, the IMRT capability of our institution was limited at that time, resulting in relatively long waiting times between the initial consultation and the initiation of IMRT. The median NA‐ADT duration was 8.3 months (range: 5.6–18.2).

After completion of WP SIB‐IMRT, adjuvant ADT (A‐ADT), consisting of an LH‐RH agonist was planned for 2 years. The median A‐ADT duration was 24.7 months (range: 0–68.2). No patient received androgen receptor axis‐targeted agents (ARAT) or chemotherapy under a hormone‐sensitive condition.

### 2.4 Patient follow‐up and salvage therapy

Following IMRT completion, patients were followed‐up at 1–3‐month intervals during the first 2 years and at 3–4‐month intervals thereafter. Salvage ADT was initiated after the completion of A‐ADT when the PSA level exceeded 4 ng/ml or when any clinical recurrence was detected. If the PSA level increased during A‐ADT, an anti‐androgen was added to the LH‐RH agonist and permanently continued.

### 2.5 Outcome evaluation and statistical analyses

The biochemical relapse‐free survival (BRFS), clinical relapse‐free survival (CRFS), prostate cancer‐specific survival (PCSS), and overall survival (OS) rates were calculated using the Kaplan–Meier estimation and the Log‐rank test from the initiation date of IMRT. Cumulative incidences of acquired castration‐resistance were also estimated, with death treated as a competing risk. Acute and late rectal and urinary toxicities were graded using the definitions of the Common Terminology Criteria for Adverse Events (CTCAE) version 4.0. Adverse events occurring within 90 days of the start date of RT were defined as acute adverse events, and those occurring after that date were defined as late adverse events. BRFS rates were evaluated based on the Phoenix definition.^13^ However, for patients whose PSA level had elevated during the A‐ADT, the date of the first rise of the PSA level was considered the biochemical failure date. In this study, a castration‐resistant status was defined as a PSA increase in a monotonic manner not only under castrated levels of testosterone but also under ADT or post‐surgical castration.

Univariate analysis and multivariate analysis was planned to find predictive and prognostic factors related to clinical outcomes. Factors included age, performance status (PS), pretreatment PSA level, clinical T stage (T3b–T4 vs. T1–T3a), GG (group 5 vs. 1–4), and a posttreatment nadir PSA level (undetectable level [<0.01 ng/ml] or not). If the total number of patients and events were not enough for multivariate analysis, we performed only univariate analyses using the Cox‐proportional hazard model. Patients who were lost to follow‐up, due to castration‐resistant diseases, were classified as “dead from prostate cancer” at the point of the last visit. A value of *p* < 0.05 was considered to indicate statistical significance. All statistical analyses were conducted using EZR software (Saitama Medical Center, Jichi Medical University, Saitama, Japan), a graphical user interface for R (R Foundation for Statistical Computing).

## 3 RESULTS

### 3.1 Oncological and survival outcomes

**FIGURE 1:**
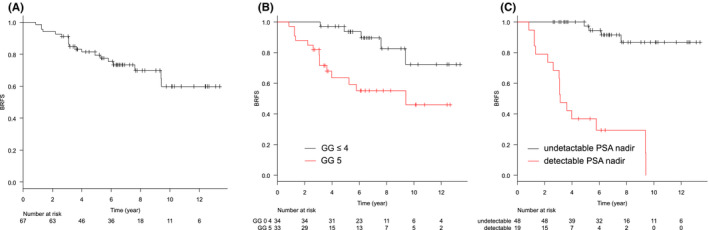
Kaplan–Meier curves of BRFS in (A) all patients, (B) the patients with GG ≤4 or 5, and (C) the patients with detectable PSA level or not. BRFS, biochemical failure‐free survival; GG, grade group; PSA, prostate‐specific antigen

**FIGURE 2:**
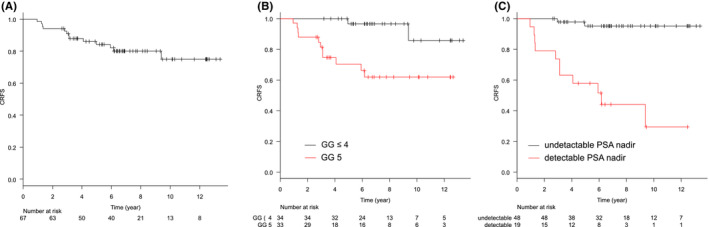
Kaplan–Meier curves of CRFS (A) in all patients, (B) the patients with GG ≤4 or 5, and (C) the patients with detectable PSA level or not. CRFS, clinical relapse‐free survival; GG, grade group; PSA, prostate‐specific antigen

**FIGURE 3:**
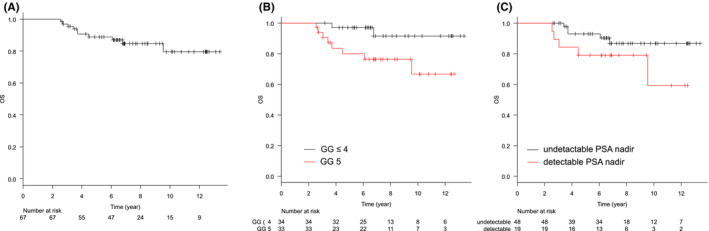
Kaplan–Meier curves of OS in (A) all patients, (B) the patients with GG ≤4 or 5, and (C) the patients with detectable PSA level or not. GG, grade group; OS, overall survival; PSA, prostate‐specific antigen

**FIGURE 4:**
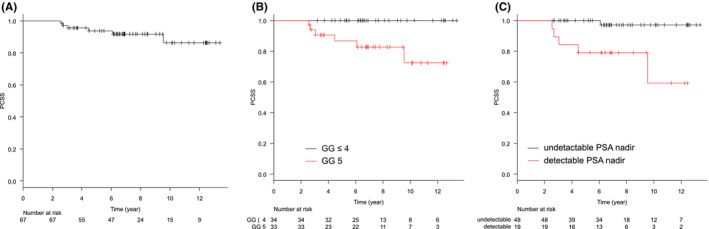
Kaplan–Meier curves of PCSS in (A) all patients, (B) the patients with GG ≤4 or 5, and (C) the patients with detectable PSA level or not. GG, grade group; PCSS, prostate cancer‐specific survival; PSA, prostate‐specific antigen

**FIGURE 5:**
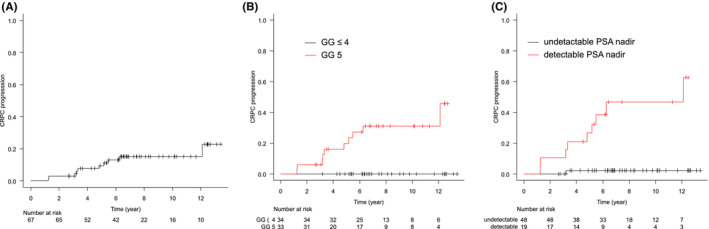
Cumulative incidence rates for castration‐resistant status in (A) all patients, (B) the patients with GG ≤4 or 5, and (C) the patients with detectable PSA level or not. GG, grade group; PSA, prostate‐specific antigen

The median follow‐up period was 81.6 months (range: 30.5–160.7). Treatment details are summarized in Table 2. BRFS and CRFS rates were, respectively, 79.4% and 84.2% at 5 years, and 59.8% and 75.0% at 10 years (Figures 1 and 2). Clinical recurrence was observed in 13 patients, and the first site of recurrence was local in 1, nodal in 5, skeletal in 4, lung in 1, and nodal and skeletal in 1. In one patient, antiandrogen therapy was started at the physician's discretion before the patient met the definition of biochemical recurrence, and the initiation date of the therapy was defined as the date of clinical recurrence. At the time of clinical recurrence, none of the patients presented with symptoms associated with recurrent disease. Log‐rank tests showed that the pretreatment PSA level, being in GG 5, and having detectable nadir PSA levels were significantly associated with worse BRFS. Being in GG 5 and having detectable nadir PSA levels were significantly associated with worse CRFS (*p* = 0.009 and *p* < 0.001, respectively). The other factors evaluated including age, PS, or T stage were not associated with BRFS or CRFS. Loco‐regional recurrence was not observed. The OS and PCSS rates were, respectively, 88.9% and 93.7% at 5 years, and 79.6% and 86.3% at 10 years (Figures 3 and 4). Log‐rank tests showed that being in GG 5 was significantly associated with worse OS (*p* = 0.049) and PCSS (*p* < 0.001). A detectable nadir PSA level was significantly associated with worse PCSS (*p* = 0.002), but not with OS (*p* = 0.111). Tables 3 and S1 show the univariate analysis of BRFS, CRFS, PCSS, and OS. The number of events was not enough for multivariate analysis. The cumulative incidence of castration resistant status at 10 years was 15.1% (Figure 5). Of the 10 patients who progressed to castration‐resistant prostate cancer (CRPC), 5 received ARAT and 5 received chemotherapy after CRPC progression. Being in GG 5 and having an detectable nadir PSA level were significantly correlated to CRPC progression (both *p* < 0.001). No patient with GG ≤4 disease progressed to CRPC, and only 1 of 48 patients with undetectable nadir PSA levels progressed to CRPC.

**TABLE 2:** Summary of treatments

Neoadjuvant ADT	Contents	CAB	65
		Orchiectomy	2
		None	0
	Duration (months)	Median	8.3
		Range	5.6–18.2
Adjuvant ADT	Duration (months)	Median	24.7
		Range	0–68.2
Radiation	Total dose	78 Gy	59
		74 Gy	8

Abbreviations: ADT, androgen deprivation therapy; CAB, combined androgen blockade.

**TABLE 3:** Univariate Cox proportional hazards regression analyses for BRFS and PCSS

	BRFS			PCSS		
	HR	95% CI	*p*‐value	HR	95% CI	*p*‐value
Age (per year)	0.9698	(0.8985–1.047)	0.430	1.005	(0.8784–1.149)	0.946
PS						
0	1.0	(ref.)		1.0	(ref.)	
1	0.301	(0.04011–2.258)	0.243		NA^a^	
Pretreatment PSA (ng/ml)						
≤20	1.0	(ref.)		1.0	(ref.)	
>20	4.422	(1.019–19.19)	0.047	0.9451	(0.173–5.164)	0.948
ISUP Grade group						
≤4	1.0	(ref.)		1.0	(ref.)	
5	4.025	(1.444–11.22)	0.008		NA^a^	
Clinical T stage						
T2–T3a	1.0	(ref.)		1.0	(ref.)	
T3b–T4	1.605	(0.6284–4.101)	0.327	1.765	(0.32–9.734)	0.514
PSA nadir						
<0.01	1.0	(ref.)		1.0	(ref.)	
≥0.01	18.15	(5.912–55.72)	<0.001	13.38	(1.561–114.7)	0.018

Abbreviations: BRFS, biochemical relapse‐free survival; CI, confidence interval; HR, hazard ratio; ISUP, International Society of Urological Pathology; PCSS, prostate cancer‐specific survival; PS, performance status; PSA, prostate‐specific antigen; ref, reference.

^a^ Univariate analysis could not be performed due to no events occurring in the reference or evaluation groups.

### 3.2 Acute and late toxicity

Based on the CTCAE version 4.0, the crude incidence rates of grade 2 and 3 acute toxicities were, respectively, 28.4%, and 0% for genitourinary events, and 7.5% and 0% for gastrointestinal events. The cumulative incidence rates of grade 2 and 3 late genitourinary toxicities were, respectively, 1.5% and 0% at 5 years, and also 1.5% and 0% at 10 years. The cumulative incidence rates of grade 2 and 3 late gastrointestinal toxicities were, respectively, 0% and 1.5% at 5 years, and also 0% and 1.5% at 10 years. Late adverse events included one case of grade 2 urinary frequency requiring alpha blockers and one case of grade 3 rectal bleeding requiring hemostasis with argon plasma coagulation. No grade 4 or higher acute or late toxicities were observed.

## 4 DISCUSSION

Most malignant diseases with regional node metastases are considered curable. Therefore, they are categorized as different entities from those with distant metastasis and are treated definitively (e.g., head and neck, lung, cervical, and colorectal cancer). However, the current Union for International Cancer Control staging system categorizes N1M0 prostate cancer as stage IV, the same as prostate cancer with distant metastasis. NCCN guidelines^2^ recommend either long‐term ADT (with or without abiraterone) with or without radiation therapy with ADT, or radical prostatectomy for N1M0 prostate cancer patients. Therefore, many N1M0 prostate cancer patients are treated with lifelong ADT, which is not considered a curative treatment. Moon et al.^14^ reported that ADT alone was the most common treatment (46.0%) for N1M0 prostate cancer patients from 2006 to 2011 in the United States. One of the reasons for this was the rarity of N1M0 prostate cancer and lack of prospective trials comparing ADT alone versus ADT with RT. However, recent multiple retrospective studies and database analyses showed the efficacy of local treatment (prostatectomy or EBRT) for N1M0 prostate cancer patients.^10^, ^15^, ^16^

For EBRT without pelvic irradiation in clinically node‐negative prostate cancer patients, a meta‐analysis showed that increasing the radiation dose up to 84 Gy significantly improves BRFS.^6^ On the other hand, for EBRT with pelvic irradiation, radiation to the prostate using conventional techniques could not be increased due to the dose limitations of the rectum and bladder. Published phase III trials comparing prostate‐only EBRT and WP EBRT with <70 Gy showed a marginally significant increase in late gastrointestinal grade ≥3 toxicity in WP EBRT groups.^17^, ^18^, ^19^, ^20^ IMRT could realize dose escalation to the prostate without increasing the dose to normal tissue in a WP irradiation setting. In our study, the incidence rates of late grade ≥3 genitourinary and gastrointestinal toxicities were 0% and 1.5%, respectively, which are less than those of conventional WP EBRT trials.^17^, ^18^, ^19^, ^20^

Few reports on WP EBRT treatment of patients with N1M0 prostate cancer have been published. One cohort study extracted from a STAMPEDE, prospective, multi‐arm, multistage platform designed trial, comparing ADT versus ADT + RT was reported.^21^ The reported failure‐free survival rates at 2 years were 81% and 53%, respectively, in the ADT + RT and ADT‐alone groups, and the adjusted hazard ratio was 0.48. This was the first and only prospective trial demonstrating the effectiveness of RT for N1M0 prostate cancer, as far as we know. However, application of IMRT was not mandatory in this trial and 18.3% (13/71) of patients in the ADT + RT group received prostate‐only RT instead of WP RT. Therefore, this trial showed the efficacy of local treatment for N1M0 prostate cancer patients, but might have underestimated the efficacy of WP IMRT for such conditions.

Our study found that the clinical T stage and pretreatment PSA levels were not correlated with PCSS, but the GG and nadir PSA levels were found to be significantly correlated with the outcomes. Several reports on the relationship between nadir PSA levels and PCSS were published^22^, ^23^, ^24^; however, they analyzed N0M0 and distant‐metastatic cases. This is the first article regarding N1M0 cases. In previous reports, authors divided groups at 0.2 ng/ml for nadir PSA because the lower limit of sensitivity at the time of the report was 0.2 ng/ml. In the present report, we also aimed to evaluate undetectable versus detectable nadir PSA group, and defined a nadir PSA of less than 0.010 ng/ml as the undetectable group. Although some facilities were capable of measuring up to 0.008, we defined the lower detectable limit as 0.010 ng/ml, for the purpose of consistency.

Regarding CRPC progression, both nadir PSA and GG 5 are related to CRPC progression. In fact, all 10 patients who progressed to CRPC had GG 5 disease, therefore further increase of treatment intensity (e.g., earlier administration of ARAT) could be considered for GG5 cases. When grouped by nadir PSA, the undetectable group consisted of 48 patients, only one of whom progressed to CRPC. Conversely, 9 of the 19 cases in the detectable PSA group progressed to CRPC. This result suggests that patients whose PSA does not decrease less than 0.010 ng/ml are at high risk of developing hormonal resistance. Therefore, if PSA does not decrease to an undetectable level after pelvic irradiation with CAB not ARAT, additional therapy (ARAT or chemotherapy) should be considered early.

In 2006 when we started WP‐SIB‐IMRT for cN1M0 prostate cancer patients, the standard dose fractionation was 1.8–2 Gy per fraction for prostate cancer. Therefore, in the protocol for N1M0 prostate cancer, we decided to irradiate the prostate in 2‐Gy fractions. At the same time, SIB‐IMRT but not 2‐step IMRT was applied, and the LN metastasis and the lymphatic region were irradiated in 1.7‐ and 1.5‐Gy fraction, respectively. The alpha beta value is thought to be about 1.5 Gy^25^; therefore, the 1.7‐ and 1.5‐Gy fractionation in the protocol is not ideal for prostate cancer treatment based on the linear‐quadratic model. However, it is worth noting that the incidence of toxicities is very low, and no recurrence in the prophylactic irradiated region has been observed. Recently, moderately hypofractionated IMRT (2.5–3 Gy per fraction) has become one of the standard treatment options for localized prostate cancer, and moderately hypofractionated WP irradiation has been partially applied in some prospective trials.^20^, ^26^ We expect that moderate hypofractionation will be more widely applied in WP irradiation. In addition, short‐term results of ultrahypofractionation, including prophylactic irradiation to the pelvis, have been reported.^27^ Although the median follow‐up period was short (30 months), the reported BRFS and OS were good (77% and 89%, respectively), and depending on the long‐term results, ultrahypofractionation may become the standard for pelvic irradiation as well.

Our report is, to the best of our knowledge, the first study conducted with homogeneous patient and treatment characteristics. This is paired with the study having the longest follow‐up period of WP IMRT for N1M0 prostate cancer patients reported to date. However, our study has several limitations. The first is its retrospective design. We treated the patients according to a predefined protocol, but selection bias must be considered. Second, the diagnoses of positive pelvic node and negative distant metastasis were not confirmed by prostate specific membrane antigen positron emission tomography, ultrasmall superparamagnetic iron oxide MRI, or diffusion‐weighted whole‐body imaging with background suppression. Third, NA‐ADT consisted of CAB in most cases. While this is very common in Japan, in Europe, and the United States an LH‐RH agonist alone is used. Despite these limitations, our data suggest potential benefits from ADT + WP‐IMRT and predict some risk factors for N1M0 patients.

In conclusion, WP SIB‐IMRT can be delivered safely to patients with pelvic node‐positive prostate cancer. Clinical outcomes seem promising, although the number of patients and the follow‐up period are limited in the present study. Both GG 5 and a detectable nadir PSA were significant risk factors for prostate cancer death in N1M0 patients; therefore, we may need to study the effects of increasing the intensity of treatment for such cases prior to CRPC progression.

## AUTHOR CONTRIBUTIONS

**Kiyonao Nakamura:** Conceptualization (lead); data curation (lead); formal analysis (lead); investigation (lead); methodology (lead); project administration (lead); resources (equal); software (lead); visualization (lead); writing – original draft (lead). **Yoshiki Norihisa:** Conceptualization (supporting); investigation (supporting); methodology (supporting); supervision (supporting); writing – review and editing (supporting). **Itaru Ikeda:** Conceptualization (supporting); investigation (supporting); supervision (supporting); writing – review and editing (supporting). **Haruo Inokuchi:** Conceptualization (supporting); investigation (supporting); supervision (supporting); writing – review and editing (supporting). **Rihito Aizawa:** Investigation (supporting); methodology (supporting); writing – review and editing (supporting). **Toshiyuki Kamoto:** Conceptualization (supporting); investigation (supporting); methodology (supporting); supervision (supporting); writing – review and editing (supporting). **Tomomi Kamba:** Investigation (supporting); methodology (supporting); supervision (supporting); writing – review and editing (supporting). **Takahiro Inoue:** Investigation (supporting); methodology (supporting); supervision (supporting); writing – review and editing (supporting). **Toshinari Yamasaki:** Investigation (supporting); supervision (supporting); writing – review and editing (supporting). **Shusuke Akamatsu:** Investigation (supporting); supervision (supporting); writing – review and editing (supporting). **Takashi Kobayashi:** Investigation (supporting); supervision (supporting); writing – review and editing (supporting). **Osamu Ogawa:** Conceptualization (supporting); investigation (supporting); methodology (supporting); supervision (supporting). **Takashi Mizowaki:** Conceptualization (equal); funding acquisition (lead); investigation (supporting); methodology (supporting); supervision (lead); writing – review and editing (supporting).

## FUNDING INFORMATION

This work was supported, in part, by JSPS KAKENHI Grant Number JP22H03022. The funding programs had no effects on the study design, analysis, and interpretation of the data of this manuscript.

## CONFLICT OF INTEREST

Toshiyuki Kamoto has received honoraria from AstraZeneca, Sanofi, and Janssen Pharmaceutical, and grant support from Taiho Pharmaceutical Company Limited, Nippon Shinyaku Company Limited, and Chugai Pharmaceutical Company Limited. Tomomi Kamba has received honoraria from AstraZeneca and Merck Biopharma Company Limited. Takashi Mizowaki has received honoraria from Varian Medical Systems, Hitachi Limited, Elekta, and BrainLab, grant support from Varian Medical Systems, Hitachi Limited, and Brainlab, and consulting fees from Varian Medical Systems.

## Supporting information


Table S1.

## Data Availability

Research data are not shared.
