# Supplementary material for: Ten‐year outcomes of whole‐pelvic intensity‐modulated radiation therapy for prostate cancer with regional lymph node metastasis
Source: Cancer Med. 2022 Dec 19;12(7):7859–67. doi: 10.1002/cam4.5554 (PMC10134326; doi:10.1002/cam4.5554)
Supplement: Supplementary file 1 — Table S1. [file CAM4-12-7859-s001.docx]

Supplement

|  | CRFS |  |  |  | OS |  |  |
| --- | --- | --- | --- | --- | --- | --- | --- |
|  | HR | 95% CI | p-value |  | HR | 95% CI | p-value |
| Age (per year) | 1.032 | (0.9421–1.131) | 0.495 |  | 1.05 | (0.9425–1.169) | 0.377 |
| PS |  |  |  |  |  |  |  |
| 0 | 1.0 | (ref.) |  |  | 1.0 | (ref.) |  |
| 1 | 0.4728 | (0.06131–3.646) | 0.472 |  | 0.6693 | (0.0844–5.308) | 0.704 |
| Pretreatment PSA (ng/ml) |  |  |  |  |  |  |  |
| ≤20 | 1.0 | (ref.) |  |  | 1.0 | (ref.) |  |
| >20 | 1.524 | (0.4188–5.543) | 0.523 |  | 0.6804 | (0.1918–2.414) | 0.551 |
| ISUP Grade Group |  |  |  |  |  |  |  |
| ≤4 | 1.0 | (ref.) |  |  | 1.0 | (ref.) |  |
| 5 | 7.372 | (1.629–33.36) | 0.009 |  | 4.763 | (1.01–22.47) | 0.049 |
| Clinical T stage |  |  |  |  |  |  |  |
| T2-T3a | 1.0 | (ref.) |  |  | 1.0 | (ref.) |  |
| T3b-T4 | 2.078 | (0.6368–6.77) | 0.226 |  | 0.8661 | (0.2496–3.005) | 0.821 |
| PSA nadir |  |  |  |  |  |  |  |
| <0.01 | 1.0 | (ref.) |  |  | 1.0 | (ref.) |  |
| ≥0.01 | 18.06 | (3.992–81.71) | <0.001 |  | 2.641 | (0.7638–9.131) | 0.125 |

Abbreviations: CRFS, clinical relapse-free survival; OS, overall survival; HR, hazard ratio; CI, confidence interval; PS, performance status; PSA, prostate-specific antigen; ref, reference

†: Univariate analysis could not be performed due to no events occurring in the reference or evaluation groups.
